# Allogeneic cell-based immunotherapy combined with chemotherapy and targeted therapy in advanced pancreatic cancer with metastases: A case report

**DOI:** 10.3892/ol.2014.1908

**Published:** 2014-02-24

**Authors:** YANYAN LONG, QIONG SUN, JIANYU WU, YU WANG, SHUNCHANG JIAO

**Affiliations:** 1 Department of Medical Oncology, Chinese PLA General Hospital, Beijing 100853, P.R. China; 2 Medical School of Nankai University, Tianjin 300071, P.R. China; 3 Beijing ImmunoTech Applied Science Limited, Beijing 101111, P.R. China

**Keywords:** expanded activated allogeneic lymphocytes, expanded activated autologous lymphocytes, pancreatic cancer, adoptive immunotherapy, chemotherapy, nimotuzumab

## Abstract

Immunotherapy may be an effective and potentially less toxic treatment for cancer in addition to the traditional therapies. The current study presents a case of advanced pancreatic cancer that was treated with cell-based immunotherapy using expanded activated allogeneic lymphocytes (EAAL^*^) *in vitro* with cluster of differentiation (CD)3(+) and CD8(+) cytotoxic T lymphocytes, and CD3(−) and CD56(+) natural killer cells as the major effector cells, together with chemotherapy and targeted agents. A 46-year-old female was diagnosed at the Chinese PLA General Hospital (Beijing, China) with stage IV pancreatic cancer with multiple metastases in October 2012. After receiving one cycle of chemotherapy plus nimotuzumab (Nimo), the patient received 14 infusions of EAAL^*^, which was obtained from a related donor, combined with seven cycles of chemotherapy with gemcitabine plus oxaliplatin and targeted therapy with Nimo. The patient was followed up for eight months. One day prior to the cell infusion, targeted therapy was administered and 48 h following the cell infusion, chemotherapy was administered. Following this treatment, carbohydrate antigen 19-9 levels decreased from 4,136 U/ml to within the normal ranges, along with the significant regression of the lesions. Occasionally mild upset was observed following the EAAL^*^ transfusion. For the entire combined modality, grade II hematological and gastrointestinal toxicities plus grade I liver function damage and skin rash were identified. The present study demonstrated that combining allogeneic cell-based immunotherapy with conventional therapies is effective and safe, even in patients with end-stage pancreatic cancer. Therefore, this strategy is recommended for the treatment of similar cases.

## Introduction

Pancreatic ductal adenocarcinoma (PDA), also known as pancreatic cancer, is the fourth leading cause of cancer-related mortality in the United States (1). Survival has not markedly improved despite the routine use of surgery, chemotherapy and radiotherapy. The overall five-year survival rate is <5% and the median overall survival is less than six months (1,2). In addition, only <20% of patients present with potentially curable localized resectable tumors (3). However, the majority of patients are likely to develop local recurrence or metastasis following surgery. For patients with metastatic disease, PDA is lethal and notoriously difficult to treat and such individuals exhibit a poor median survival of three to six months (4). Over the past decade, gemcitabine (Gem)-based chemotherapy or chemoradiation have been the standard regimen, although, the overall therapeutic efficacy of these methods is considered to be minimal (5,6). In order to improve the current treatment status to achieve greater efficacy and to improve prognosis, novel treatment strategies must be investigated.

Multiple new agents with diverse mechanisms of action in combination with Gem have been previously assessed in randomized clinical trials of pancreatic cancer, with no improvement in outcome observed (2,7,8). To date, the laboratory results of targeted therapies have been significant and only erlotinib, an epidermal growth factor receptor (EGFR)-tyrosine kinase inhibitor, has achieved a modest survival benefit in combination with Gem in a previous phase III clinical trial (9). Nimotuzumab (Nimo) is a humanized monoclonal antibody that recognizes the EGFR extracellular domain. Based on the results of previous phase I/II trials for pancreatic cancer, the recommended dose of Nimo has been established at 200 mg per week. In addition, Nimo is safe and well tolerated, although, the efficacy of monotherapy is minimal. At present, a randomized, placebo-controlled trial of Gem plus Nimo has been initiated, of which the results are of interest (10).

Immunotherapeutic approaches are becoming promising strategies for effectively inducing antitumor immune responses with reduced toxicity (11–13). However, the manner in which immunotherapy may be optimally integrated with existing non-immunological therapies for optimal synergy remains to be elucidated (14). In addition, an approach should be established to arrange the order of the various combination treatments, including immunotherapy, chemotherapy and monoclonal antibodies (15–18).

Expanded activated autologous lymphocyte (EAAL) therapy with cluster of differentiation (CD)3(+) and CD8(+) cytotoxic T lymphocytes, and CD3(−) and CD56(+) natural killer cells as the major effector cells is a type of adoptive cell therapy. It has previously been shown that EAAL therapy has the ability to enrich potential antitumor responses and that it is safe for early- and late-stage cancer patients (19,20). A randomized trial sponsored by Takayama *et al* (21) demonstrated that adoptive immunotherapy lowers postsurgical recurrence rates of hepatocellular carcinoma with significantly longer recurrence-free (P=0.01) and disease-specific (P=0.04) survival than those of the control group. Expanded activated allogeneic lymphocyte (EAAL^*^) therapy is a type of EAAL therapy with infusion lymphocytes, which are obtained from a human leukocyte antigen (HLA)-matched related donor rather than from the patients themselves.

The present study reports the eight-month follow-up of a patient with advanced pancreatic cancer with multiple metastases. The patient was treated with EAAL^*^ therapy obtained from a related donor in addition to conventional chemotherapy with Gem and oxaliplatin (L-OHP) plus targeted therapy with Nimo. Written informed consent was obtained from the family of the patient.

## Case report

A 46-year-old female presented to with cough and expectoration with no apparent cause in October 2012 at the local doctor. Positron emission tomography (PET)/computed tomography (CT) and biopsy revealed a PDA involved in the body of the pancreas with multiple metastases to the lungs, liver and abdominal lymph nodes. The carbohydrate antigen (CA) 19-9 value was 3,318 U/ml at diagnosis. Prior to the cell-based immunotherapy, the patient received one cycle of intravenous chemotherapy with 1,800 mg Gem (1,000 mg/m^2^ i.v. on days one and eight, every 21 days) and 150 mg L-OHP (85 mg/m^2^ i.v. on day one, every 21 days), and targeted therapy with 200 mg Nimo (i.v. on day seven, every seven days). The immunotherapy was subsequently initiated. At diagnosis, the tumor load of the patient was considered to be large, due to multiple metastases, and the patient had relatively weak immunity, thus, the EAAL^*^ therapy was designed (Beijing ImmunoTech Applied Science Ltd., Beijing, China). Written informed consent was obtained and the patient’s HLA genotype was matched with that of a related donor, peripheral blood was collected from the related donor in heparin tubes and transported to the laboratory under cold conditions.

Activated lymphocytes using anti-CD3 monoclonal antibody and interleukin-2 were generated as described previously (22). Briefly, 20–100 ml of peripheral blood was collected from the related donor and peripheral blood mononuclear cells (PBMCs) were isolated by Ficoll-Hypaque gravity centrifugation (ALLEGRA X-12, Beckman Coulter, Miani, FL, USA) at 400 × g. The isolated PBMCs were washed and resuspended in serum-free medium (IMSF 100; Immunotech, London, UK) supplemented with 700 U/ml of interleukin (IL)-2 (CCBIO, Changchun, China). The PBMC suspension was placed in a flask coated with immobilized anti-CD3 antibody (eBioscience, San Diego, CA, USA)and incubated for one week. The lymphocyte suspension was transferred to a gas-permeable bag to allow the lymphocytes to grow for two more weeks. The activated lymphocytes were subsequently harvested, filtered through 100-μm membranes and resuspended in 100 ml of normal saline containing 1% human serum albumin for the intravenous infusion. Prior to cell transplantation, the cells were assessed for endotoxin levels using a Limulus Amebocyte Lysate kit (Associates of Cape Cod, Inc., Falmouth, MA, USA). The average cell count following the *in vitro* expansion was 2.04–5.18×10^9^ cells/100 ml. Therefore, the patient was administrated 100 or 200 ml (large dose) of these activated lymphocytes up to 14 times a week or every other week. For each infusion, one part of these cells was recovered, activated and expanded for two weeks and transferred into the patient as previously described.

Overall, the patient received 14 infusions of EAAL^*^, with an overall cell count of 7.414×10^10^, in combination with seven cycles of chemotherapy plus targeted therapy. The initial four infusions of EAAL^*^ were at doses of 100 ml and were followed by doses of 200 ml. The specific regimen was as follows: EAAL^*^, 100 ml/200 ml i.v. on days 2 and 9; Gem, 1,800 mg i.v. on days 4 and 11; L-OHP, 150 mg i.v. on day 4; and Nimo, 200 mg i.v. on days 1, 8 and 15, every 21 days. Of note, during the last two cycles of chemotherapy, the dosage of Gem was reduced to 1,600 mg and that of L-OPH was reduced to 125 mg, due to concern regarding the cumulative toxicities, although, the patient tolerated the treatments well exhibiting only grade II adverse effects.

Responses were evaluated according to the Response Evaluation Criteria in Solid Tumors (RECIST) and the toxic effects were assessed using the National Cancer Institute Common Toxicity Criteria, version 3.0 (23,24). The responses were recorded during and following each infusion and the final follow-up was in July 2013.

**Figure 1:**
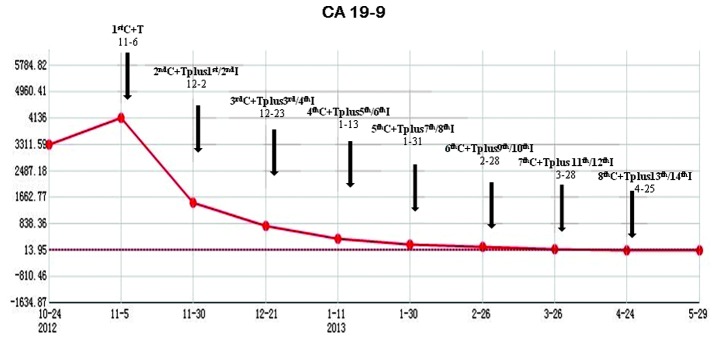
Curve demonstrating the decrease in CA 19-9 values during the whole course of treatment, with eight cycles of chemotherapy plus targeted therapy combined with 14 infusions of EAAL^*^ therapy. CA 19-9, carbohydrate antigen 19-9; EAAL^*^, expanded activated allogeneic lymphocytes; C, chemotherapy with gemcitabine and oxaliplatin; T, targeted therapy with nimotuzumab; I, EAAL^*^-based immunotherapy.

Following two infusions of EAAL^*^ in combination with one cycle of chemotherapy plus targeted therapy, the patient’s response was evaluated. A CT scan in December 2012 revealed that lesions in the pancreas, liver, lungs and abdominal lymph nodes showed slight shrinkage. In addition, the CA 19-9 levels had decreased from 4,136 U/ml (prior to treatment) to 758.50 U/ml (Fig. 1). The status of the patient was stable disease according to the RECIST criteria.

The patient continued with six infusions of EAAL^*^ combined with three cycles of chemotherapy plus targeted therapy. Responses were evaluated by CT scan, which revealed that the metastatic lesions in the lungs and liver were significantly reduced, with lesions in the pancreas being slightly reduced. In addition, the CA 19-9 levels decreased to 113.6 U/ml (Fig. 1) and partial remission (PR) was achieved.

**Figure 2:**
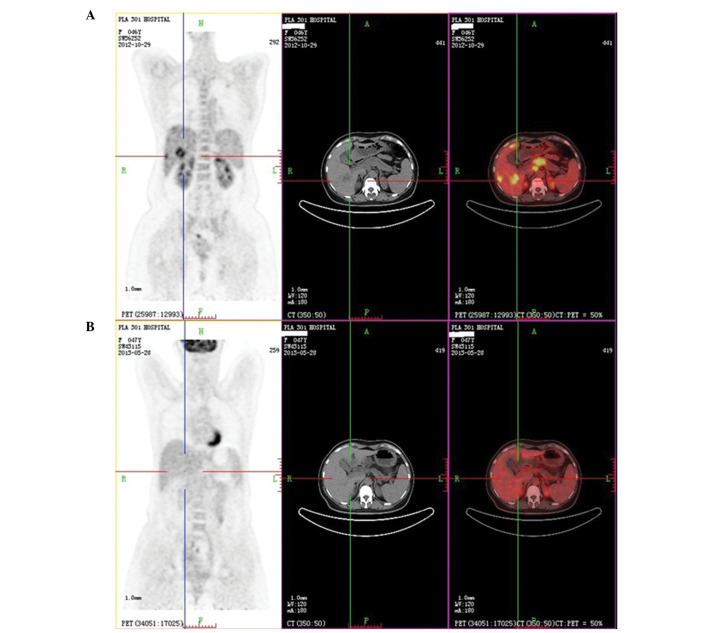
Positron emission tomography/computed tomography of the patient prior to the first cycle of chemotherapy plus targeted therapy on (A) Oct 29th, 2012 and (B) following the eighth cycle of chemotherapy plus targeted therapy combined with 14 infusions of expanded activated allogeneic lymphocyte therapy on May 28th, 2013. The metabolic values of the deposits in the pancreas, liver and abdominal lymph nodes decreased markedly and even disappeared. In addition, the deposits in the lungs decreased significantly, along with the size of all the lesions compared with the lesion sizes prior to treatment.

Finally, the patient received a further six infusions of EAAL^*^ at large doses (200 ml) together with three cycles of chemotherapy plus targeted therapy. PET/CT was performed to evaluate the responses and showed that almost all the metabolic values of the deposits had markedly decreased or even disappeared; in addition, the size of all the lesions had markedly decreased (Fig. 2). The CA 19-9 levels decreased to 24.09 U/ml (normal range, 0.1–37 U/ml; Fig. 1) and PR and near complete remission (nCR) were achieved.

The last follow-up in July 2013 revealed a static non-progressive disease with a progression-free survival (PFS) of eight months and demonstrated that all of the parameters, including the tumor markers, were within their normal ranges.

The patient showed an improved quality of life without a cough or expectoration. Following all 14 infusions of EAAL^*^, with an overall cell count of 7.414×10^10^, mild upset was occasionally identified following large-dosage lymphocyte transfusions without other severe adverse effects. For the whole combined modality, the most serious toxicities observed were grade II hematological and gastrointestinal toxicities, in addition to grade I liver function damage and a skin rash.

## Discussion

The current study presents a patient with stage IV pancreatic cancer with multiple metastases for whom curative surgery was not an option at diagnosis. A novel therapeutic strategy was administered to the patient, which included several infusions of EAAL^*^ together with Gem-based chemotherapy and Nimo-based targeted therapy. Notably, the strategy achieved an ideal and rare antitumor responses, PR and nCR. The CA 19-9 levels decreased from 4,136 U/ml to within the normal ranges. In addition, significant regression of the lesions was observed. The eight-month follow-up showed a prolonged static non-progressive disease with a PFS of seven months, which far exceeded the predictions of a previous study (4). The patient benefited from the individualized treatment and multimodality therapy, which is consistent with the previous observations that immunotherapy combined with other non-immunological therapies moderately enriches the potential antitumor responses through the mechanism(s) by which these modalities are synergized. However, these mechanisms are not fully understood (17).

Of note, the order of administration for the combined therapeutic approach is a critical factor that affects the therapeutic outcome. Therefore, establishing the order of administration to maximize the efficacy and guarantee safety is a significant problem that remains unsolved. Zhang *et al* (16) hypothesized that timely immune modification of chemotherapy-activated antitumor immunity results in an enhanced antitumor immune response and complete tumor eradication. In accordance with this, chemotherapy is commonly administered prior to autologous cell-based immunotherapy in clinical practice. However, for safety considerations, the EAAL^*^ was administered prior to chemotherapy in the current study. It was hypothesized that allogeneic cell infusion may be relatively unsafe compared with infusions of autologous lymphocytes, as it may lead to rejection or unknown adverse effects. Therefore, the current patient received chemotherapy 48 h following the allogeneic cell infusion since toxic chemotherapy agents are likely to eventually remove foreign cells. The present study showed that EAAL^*^ therapy was safe and the order of administration was effective.

To date, the patient has achieved nCR with marked regression of the deposits. Immunotherapy has been planned as a maintenance treatment to lower the local recurrence and metastasis rates, and eradicate any minimal residual disease. In addition, follow-up treatment and prognosis will be tracked for subsequent studies.

In conclusion, the current study showed that this type of combined therapy is effective and safe, therefore, we recommend that this strategy is considered for the treatment of similar cases.
